# Rice Potassium Transporter OsHAK8 Mediates K^+^ Uptake and Translocation in Response to Low K^+^ Stress

**DOI:** 10.3389/fpls.2021.730002

**Published:** 2021-08-03

**Authors:** Xiaohui Wang, Junfeng Li, Fei Li, Yu Pan, Dan Cai, Dandan Mao, Liangbi Chen, Sheng Luan

**Affiliations:** ^1^Hunan Province Key Laboratory of Crop Sterile Germplasm Resource Innovation and Application, College of Life Sciences, Hunan Normal University, Changsha, China; ^2^Department of Plant and Microbial Biology, University of California, Berkeley, Berkeley, CA, United States

**Keywords:** potassium, rice, *OsHAK8*, K^+^ uptake, K^+^ translocation, low K^+^ stress

## Abstract

Potassium (K^+^) levels in the soil often limit plant growth and development. As a result, crop production largely relies on the heavy use of chemical fertilizers, presenting a challenging problem in sustainable agriculture. To breed crops with higher K^+^-use efficiency (KUE), we must learn how K^+^ is acquired from the soil by the root system and transported to the rest of the plant through K^+^ transporters. In this study, we identified the function of the rice K^+^ transporter *OsHAK8*, whose expression level is downregulated in response to low-K^+^ stress. When *OsHAK8* was disrupted by CRISPR/Cas9-mediated mutagenesis, *Oshak8* mutant plants showed stunted growth, especially under low-K^+^ conditions. Ion content analyses indicated that K^+^ uptake and root-to-shoot K^+^ transport were significantly impaired in *Oshak8* mutants under low-K^+^ conditions. As the *OsHAK8* gene was broadly expressed in different cell types in the roots and its protein was targeted to the plasma membrane, we propose that OsHAK8 serves as a major transporter for both uptake and root-to-shoot translocation in rice plants.

## Highlights

- Potassium transporter OsHAK8 mediates K^+^ uptake and translocation under low-K^+^ stress.

## Introduction

Potassium (K^+^) is an essential macronutrient for plant growth and development. Therefore, sufficient K^+^ supplies are necessary to promote crop growth and development (Clarkson and Hanson, 1980; Leigh and Wyn Jones, 1984). Although the K^+^ concentration in the cytoplasm often reaches 100 mM, the K^+^ concentration in the soil is much lower, usually below 1 mM (Maathuis, 2009). Therefore, plants often suffer low-K^+^ stress under natural conditions, limiting their growth and development (Schroeder et al., 1994). However, plants can sense potassium levels in soil and adapt to low-K^+^ stress by altering root morphology, modifying K^+^ acquisition, and changing the K^+^ utilization strategy they utilize (Schachtman and Shin, 2007; Luan et al., 2009; Luan et al., 2017; Tang et al., 2020).

K^+^ absorption by plant roots and K^+^ transport throughout the plant are mediated by transport proteins including K^+^ channels and carriers. The activities of these K^+^ channels and carriers are highly regulated, allowing plants to respond and adapt to low-K^+^ stress (Schachtman and Shin, 2007; Luan et al., 2009; Luan et al., 2017; Wang and Wu, 2017; Tang et al., 2020). To date, many channels and carriers involved in K^+^ absorption and translocation have been identified and their functions characterized in the model plant *Arabidopsis* (Adams and Shin, 2014; Luan et al., 2017; Tang et al., 2020). For example, *AtAKT1* encodes an inward-rectifying K^+^ channel that is expressed highly in the roots and contributes to K^+^ uptake (Lagarde et al., 1996; Hirsch et al., 1998). Under low-K^+^ conditions, AtAKT1 is activated in plant root hair by a regulatory pathway, which entails calcineurin B-like calcium sensors (CBL1 and CBL9) functioning together with their interacting kinase (CIPK23) (Li et al., 2006; Xu et al., 2006; Cheong et al., 2007; Luan, 2009; Luan et al., 2009). Moreover, AtHAK5 from the HAK/KUP/KT family is characterized as a high-affinity K^+^ transporter in *Arabidopsis* roots. The transcription of *AtHAK5* is induced under K^+^-deficient conditions (Rubio et al., 2000; Gierth et al., 2005), and transport activity is also enhanced by CBL1/9-CIPK23-dependent phosphorylation (Ragel et al., 2015). AtHAK5 and AtAKT1 are considered as the two major components that contribute to K^+^ uptake in *Arabidopsis* roots (Gierth et al., 2005; Pyo et al., 2010; Nieves-Cordones et al., 2014). After being absorbed into the root hairs and epidermis, K^+^ is transported into root stele tissues and then translocated from roots toward shoots *via* xylem vessels which serve as the highway for long-distance K^+^ translocation. The outward-rectifying Shaker K^+^ channel AtSKOR is involved in this process. *AtSKOR* is expressed in stele tissues and is responsible for K^+^ release into the xylem sap (Gaymard et al., 1998). In addition to AtSKOR, the K^+^ transporter AtKUP7 may also participate in K^+^ transport into the xylem sap, affecting K^+^ translocation from roots toward shoots, especially under K^+^-limited conditions (Han et al., 2016). It is noteworthy that other transporters that are not K^+^-specific, such as Nitrate Transporter 1/Peptide Transporter AtNRT1.5, Cation Chloride Co-transporter AtCCC, and Barley Non-selective Outwardly Rectifying Current channel NORC have also been reported to facilitate K^+^ efflux into the xylem sap (Colmenero-Flores et al., 2007; Pottosin and Dobrovinskaya, 2014; Li et al., 2017).

Rice is a staple food for half of the global population and serves as a critical model for studying the basic biology of cereal crops. The growth of rice plants is highly susceptible to K^+^ deficiency; as a result, rice production requires a large amount of K^+^-fertilizers (Wanasuria et al., 1981; Dobermann et al., 1996; Li et al., 2014; Zhang et al., 2018). Therefore, effective K^+^ uptake and translocation within the organism will be the key to KUE, and dissecting the mechanism of K^+^ transport will be crucial to understanding the molecular basis for KUE. However, comparing with *Arabidopsis*, much less is known about the mechanism underlying K^+^ absorption and translocation in rice plants. Although OsAKT1 and OsHAK5, like AtAKT1 and AtHAK5, mediate K^+^ uptake in rice roots (Li et al., 2014; Yang et al., 2014), K^+^ channels such as OsKATs and OsAKTs may be involved in K^+^ accumulation in the cytoplasm in response to salt stress (Musavizadeh et al., 2021). Thus, more transporters related to K^+^ absorption and translocation need to be explored.

Compared with *Arabidopsis HAK* members, the rice genome features a large gene expansion in the *HAK* family. According to an earlier study (Yang et al., 2009) and up-to-date genomic information, we have confirmed that the genomes of the *Nipponbare* rice subspecies encode 27 OsHAK family members vs. 13 in *Arabidopsis*. The *Arabidosis AtHAK* family consists of four major clades (I–IV), whereas the rice *OsHAK* family has five clades (I–V). In particular, the singleton of *AtHAK5* clade (clade I) in *Arabidopsis* is expanded to eight members in rice (Yang et al., 2009). Therefore, mechanisms in rice are more complex, reflecting the more elaborate anatomy of the plant.

To date, 4 out of 27 *OsHAK* genes have been characterized in rice. In addition to *OsHAK5* (Li et al., 2014), *OsHAK1* and *OsHAK16* function in K^+^ uptake and translocation under a broad range of external K^+^ levels (0.05–1 mM), and *OsHAK21* confers significant potassium uptake and growth in yeasts at low K^+^ concentrations (Chen et al., 2015; Shen et al., 2015; Feng et al., 2019). In order to fully address the mechanism of K^+^ uptake and translocation in rice, we took a systematic approach in studying the function of *OsHAK* genes in rice using CRISPR/Cas9-mediated mutagenesis. Here, we showed that *OsHAK8* plays crucial role in K^+^ uptake and K^+^ translocation from root to shoot, especially under low-K^+^ conditions.

## Materials and Methods

### Plant Growth Conditions

Rice (*Oryza sativa* ssp. *japonica* “*Nipponbare*”) was used as the wild type in this study and for the development of all transgenic plant lines. Hoagland hydroponic cultivation of rice was performed according to an earlier procedure (Li et al., 2014) with modification in the concentrations of K^+^ as specified in the figure legends. Nutrient solutions were replaced once per week or as needed by experimental treatments.

### Yeast Complementation Assay

For the constructs used in functional complementation in CY162 cells, the coding sequences of *OsHAK8* were amplified using the specific primers listed in Supplementary Table 1 from rice genomic cDNA. The PCR product was digested with *Hin*dIII and *Xba*I and ligated into the pYES2 vector, and transformed into the yeast strain CY162, a K^+^ uptake-deficient strain of *Saccharomyces cerevisiae* (*MAT*α, Δ*trk1*, *trk2*:pCK64, *his3*, *leu2*, *ura3*, *trp1*, and *ade2*) (Anderson et al., 1992). We performed yeast transformations using the LiAC/ssDNA/PEG method, and the growth assays on the arginine phosphate (AP) medium were performed as described previously (Horie et al., 2011).

### qRT-PCR Analysis

Total RNA was extracted using the TRIzol reagent (Thermo Fisher Scientifiec Baltics UAB, United States) (Invitrogen) following the instructions of the manufacturer. Samples were treated with DNase (Promega) (Accurate Biotechnology (Hunan) Co., Ltd.) to eliminate genomic DNA contamination. Two micrograms of DNA-free RNA were used for reverse transcription using a Moloney murine leukemia virus reverse transcriptase (Promega) with anchored oligo (dT_18_). qRT-PCR was performed on an ABI PRISM 7,500 real-time PCR instrument (Thermo Fisher Scientific) (Applied Biosystems) using Power SYBR Green (TaKaRa) (Qiagen; CFX96 system). All the primer pairs used for the expression assays or subcloning are listed in Supplementary Table 1. Primer specificity was confirmed by analysis of the melting curves. The 2^–△△CT^ method (Livak and Schmittgen, 2001) was used to quantify the value of every sample using *Actin* as an internal reference.

### GUS Assays

The 2,000-bp fragment before the ATG codon of *OsHAK8* was amplified from rice genomic DNA (cv *Nipponbare*) with the specific primers listed in Supplementary Table 1. This fragment was used as the *OsHAK8* promoter in this study, digested with *Bam*HI and *Hin*dIII, and then constructed into the pCAMBIA1301 vector. The plasmid was transformed into *Nipponbare* rice *via* callus transformation as described previously (Upadhyaya et al., 2000), which was mediated by Agrobacterium EHA105. The transgenic lines were initially screened by hygromycin resistance and GUS staining. The histochemical analysis of GUS staining in different tissues of rice was performed as described previously (Ai et al., 2009). The samples were examined and photographed with an Olympus SZX12 microscope (Olympus, Olympus Corporation, Tokyo, Japan) equipped with a camera. The quantification of GUS activity was performed using the Image J software provided by the microscope manufacturer.

### Subcellular Localization Analysis

For subcellular localization analysis, the *OsHAK8* cDNA was amplified without its stop codon using the specific primers listed in Supplementary Table 1, subsequently digested with *Xba*I, and then inserted into the multicloning sites in the plant binary vector pCAMBIA2300 to make the 35S:*OsHAK8-GFP* fusion construct. Transient expression in rice of *OsHAK8*:*GFP* in the pCAMBIA2300 vector and *SP1:RFP* in the modified pCAMBIA1300 vector were conducted using the polyethyleneglycol method (Li et al., 2009). For the plasma membrane localization of OsHAK8, we used a confocal laser scanning microscope (LSM880, Carl Zeiss, Jena, Germany) for detection.

### Generation of *OsHAK8* CRISPR/Cas9 Knockout Transgenic Plants

The CRISPR/Cas9 system (the Cas9 vector pYLCRISPR/Cas9 Pubi-H provided by Prof. Yaoguang Liu) was applied to generate *Oshak8-1* mutants. The pYLCRISPR/Cas9 Pubi-H is a plant binary vector in which Cas9p was driven by the maize ubiquitin promoter (pUBI) with the hygromycin selectable marker gene having two *Bsa*I sites flanked by a toxic negative selectable marker (*ccd B* gene) for the cloning of the sgRNA expression cassette. The sgRNA1 sequences (GTGTGCTCTCTTTCGTGTTC**TGG**) which targeted exons2 of the OsHAK8 locus and the sgRNA2 sequences (CCCTGGTGATCATGCTGTGC**TGG**) which targeted exons9 of the *OsHAK8* locus (Supplementary Figure 1) were designed with the web tool CRISPR Plant (Xie et al., 2014). The 20-bp target sequence had to be immediately followed by an NGG motif (PAM), which is essential for Cas9 binding to the target DNA. sgRNA1 and sgRNA2 cloning into pYLCRISPR/Cas9Pubi-H was performed as described (Usman et al., 2020).

Transgenic plants were obtained by the co-culture of seed embryo-derived calli (*Oryza sativa L.* cv. *Nipponbare*) with *A. tumefaciens* strain *EHA105* (harboring the sgRNAs in the pYLCRISPR/Cas9 Pubi-H plasmids) following the procedure of Upadhyaya et al. (2000). To identify mutations in regenerated plants, genomic DNA was amplified by using mutation detection primers which were designed to flank the designated target site. Then, the sequence chromatograms were analyzed using the DSDecode tool^1^ to check the genotype of the transgenic plants. The two independent homozygous mutant lines from the T1 generation, named *Oshak8-1* and *Oshak8-2*, were used for further study. The primers used for CRISPR/Cas9 construction and mutant detection are listed in Supplementary Table 1.

### Measurement of Chlorophyll

We used the equations developed by Porra et al. (1989) to estimate chlorophyll contents. Chlorophyll was extracted from the rice shoots with 10 ml of 80% (v/v) acetone containing 2.5 mM of sodium phosphate buffer (pH 7.8) and incubated in darkness for 30 h at room temperature. After centrifugation (4°C, 15 min, 13,000 × g), we measured the absorbances A_663_._6_ and A_646_._6_ with a spectrophotometer (UV-1700, Shimadzu, Kyoto, Japan).

### Ion Content Analysis

For ion content analysis (K^+^), samples were washed thoroughly with double-distilled water three times and dried at 80°C for 48 h to constant weight. The samples were treated in a muffle furnace at 300°C for 1 h, 575°C for 7 h, and finally dissolved in 0.1-N HNO_3_. K^+^ concentrations were then measured by ICP-AES (Varian 715-ES) at the 766.49-nm wavelength (for K^+^).

### Measurement of Root K^+^ Net Uptake Rate

The measurement of the K^+^ uptake rate followed previously described procedures (Nieves-Cordones et al., 2010; Yang et al., 2014) with some modifications. Five-day-old rice seedlings were grown in hydroponic solutions containing 10 mM of K^+^ for 14 days and then transferred to 0 mM-K^+^ solutions for 5 days. For the K^+^ uptake assay, K^+^-starved plants were incubated in the medium containing 0.01 or 10 mM of K^+^ for 3 days, and the roots and shoots were collected for K^+^ content measurements using inductively coupled plasma (ICP) analysis. The net K^+^ uptake rate into the plant was calculated according to the equation (Nieves-Cordones et al., 2010): Net uptake rate = (C_2_ – C_1_)/[(t_2_– t_1_) × (R_2_ + R_1_)/2], where C is the total K^+^ content, R is the root dry weight, and t is the time; the subscripts 1 and 2 indicate the start and end of the period for which the uptake rate is calculated: t_2_ – t_1_ = 3 days, and (R_2_ + R_1_)/2 = the mean root dry weight.

### Measurement of K^+^ Export Rate in Xylem Sap

Seven-day-old rice seedlings were grown in the hydroponic solutions containing 10 mM of K^+^ for 14 days and then transferred to the medium containing 0.01 or 10 mM of K^+^ for 14 days. The detailed protocols for the shoot excision, collection of the xylem sap by cotton ball, determination of K^+^ concentration by ICP, and calculation of the xylem K^+^ export rate were described previously by Yang et al. (2014).

## Results

### Rice *Oshak8* Mutants Are Sensitive to Low K^+^ Stress

**FIGURE 1:**
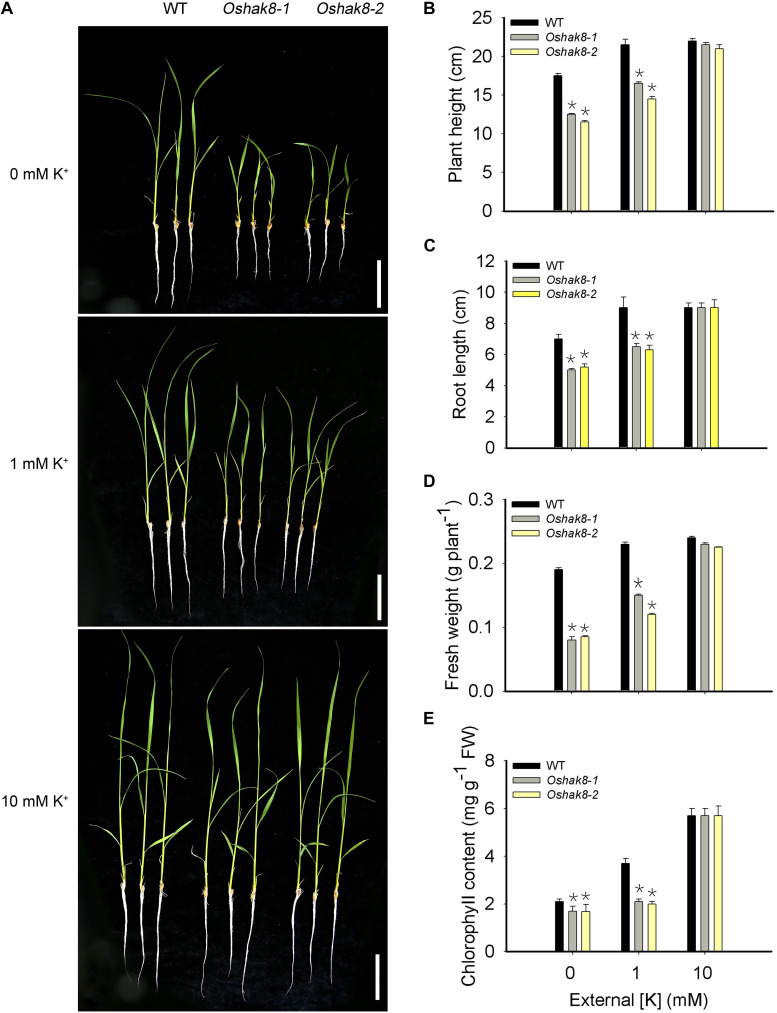
Growth retardation of the *Oshak8* mutants under K^+^-deficient conditions. **(A)** Growth retardation of the *Oshak8* mutants under K^+^-deficient conditions. The seeds of the wild-type plants (WT) and *Oshak8* mutants (*Oshak8-1*, *Oshak8-2*) germinated in water for 5 days, grown in hydroponic solutions containing 0, 1, or 10 mM of K^+^, and then photographed after 14 days. *Oshak8* mutants (*Oshak8-1*, *Oshak8-2*) displayed growth retardation under the K^+^-deficient conditions. Bars = 4 cm. **(B)** Plant height of WT and *Oshak8* mutants under various K^+^ concentrations. Root lengths of 14-day-old seedlings were measured. Growth conditions were as described in **(A)**. **(C)** Root lengths of WT and *Oshak8* mutants. Growth conditions were as described in **(A)**. **(D)** Fresh weight of WT and *Oshak8* mutants. Growth conditions were as described in **(A)**. **(E)** Chlorophyll contents of WT and *Oshak8* mutants. Growth conditions were as described in **(A)**. Significant differences were found between WT and *Oshak8* knockout plants (*Oshak8-1*, *Oshak8-2*) (*P < 0.01 by Student’s *t*-test). Data are means of three replicates of one experiment. The experiment was repeated three times with similar results. Error bars represent ± SD. Asterisks represent significant differences.

To explore the possible role of HAK family transporters in rice, we analyzed expression pattern and subcellular localization and generated gene-edited loss-of-function lines for each of the HAK members. Here, we focused on the functional analysis of OsHAK8. We produced two knockout lines of *OsHAK8* using the CRISPR/Cas9 system in the *Nipponbare* background. The *Oshak8-1* had a 1-bp insertion in the second exon of *LOC_Os03g21890*, resulting in frameshift mutations at the 68th amino acid and premature translation termination at the 87th amino acid. The *Oshak8-2* mutant had a 2-bp deletion in the 9th exon of *LOC_Os03g21890*, resulting in a frameshift mutation at the 444th amino acid (Supplementary Figure 1). No off-target cleavage was detected by the web-based tool CRISPR-P2^2^ (Liu et al., 2017). We examined the growth of the two independent *Oshak8* mutants (*Oshak8-1*, *Oshak8-2*) under K^+^-sufficient and K^+^-deficient conditions. When grown in the K^+^-sufficient hydroponic solution (10-mM K^+^), the *Oshak8* mutants and the wild-type plants (WT) grew well without discernible differences (Figure 1A). When treated in K^+^-deficient hydroponic conditions (0-mM K^+^) however, the *Oshak8* mutants showed more stunted statures than the wild-type plants. Along with the increase of external K^+^ concentrations, the differences between the *Oshak8* mutants and the wild-type plants became smaller and eventually disappeared at 10-mM K^+^ concentration (Figure 1A).

As a more quantitative measure, we assayed plant height and root length in the wild-type and mutant plants. The *Oshak8* mutants were more severely inhibited compared with the wild-type plants when grown in hydroponic conditions containing less than 10-mM K^+^ (Figures 1B,C). Consistent with the observed size difference, the fresh weights of the *Oshak8* mutants were significantly lower compared with the wild-type plants (Figure 1D). The *Oshak8* mutants also had significantly lower levels of chlorophyll than the wild-type plants (Figure 1E).

In addition, we examined the growth of the *Oshak8* mutants in soil (with 0.18 mg/g of external K^+^) (Supplementary Figure 2Aa). The *Oshak8* mutants showed necrotic brown spots and burnt tips on older leaves, which are typical K^+^-deficient symptoms of rice plants (Supplementary Figure 2Ab). The mutants were also shorter than the wild-type plants (Supplementary Figure 2B). The seed-setting rate and 1,000-grain weight in the mutants were reduced as compared to the wild-type plants (Supplementary Figures 2D,E). The main panicle length of the *Oshak8* mutants and the wild-type plants showed no discernible differences (Supplementary Figures 2Ac,C). The pollen grains in the mutants contained less starch and were less viable than those in the wild-type plants (Supplementary Figures 3A,B). These results suggest that the knockout of the *OsHAK8* gene affected rice growth and development, especially under low-K^+^ conditions, possibly due to changes in K^+^ accumulation in the mutant plants.

### Knockout of *OsHAK8* Impaired K^+^ Accumulation in Rice Plants

**FIGURE 2:**
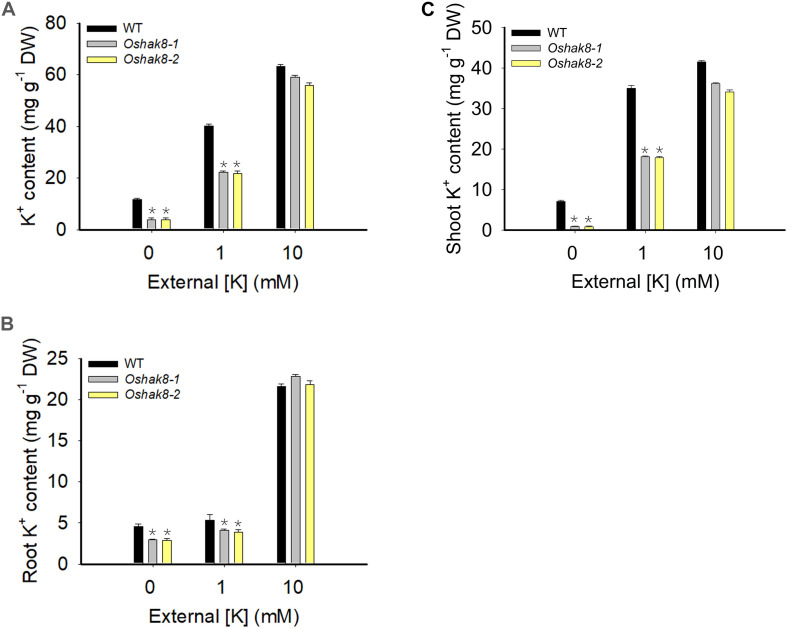
Growth retardation of the *Oshak8* mutants under K^+^-deficient conditions. The seeds of the wild-type plants (WT) and *Oshak8* mutants (*Oshak8-1*, *Oshak8-2*) germinated in water for 5 days, then grown in hydroponic solutions containing 0, 1, or 10 mM of K^+^ for 14 days. The whole plants, roots, and shoots were collected for K^+^ content measurements using inductively coupled plasma analysis. **(A)** K^+^ contents of WT and *Oshak8* mutant (*Oshak8*-1, *Oshak8*-2) plants. **(B)** K^+^ contents in the roots of WT and *Oshak8* mutant (*Oshak8*-1, *Oshak8*-2) plants. **(C)** K^+^ contents in the shoots of WT and *Oshak8* mutant (*Oshak8*-1, *Oshak8*-2) plants. Significant differences were found between WT and *Oshak8* mutant plants (*Oshak8-1*, *Oshak8-2*) (*P < 0.01 by Student’s *t*-test). Data are means of three replicates of one experiment. The experiment was repeated three times with similar results. Error bars represent ± SD. Asterisks represent significant differences.

To test whether the growth retardation phenotype of *Oshak8* was due to the reduction of K^+^ content, we measured the K^+^ content in the wild-type plants and the *Oshak8* mutants. As shown in Figure 2A, there was no significant difference in K^+^ content among the different plants when grown in the K^+^-sufficient hydroponic solution (10-mM K^+^). As the K^+^ level in the medium became lower, all plants accumulated less K^+^ as expected. However, the *Oshak8* mutants were affected more severely. For example, under 0 mM of K^+^, the *Oshak8* mutants contained 67% less K^+^ compared with the wild-type plants. Under 1 mM of K^+^, the *Oshak8* mutants accumulated 44% less K^+^ than the wild-type plants.

Then, we measured the K^+^ contents in roots and shoots separately to examine the distribution of K^+^ in the plants. As shown in Figures 2B,C, there is no difference in K^+^ content among different plants under K^+^-sufficient conditions. When grown in low-K^+^ conditions, the *Oshak8* mutants contained less K^+^ in both roots and shoots compared with the wild-type plants. We conclude from the above data that the knockout of *OsHAK8* impaired K^+^ accumulation in the plants, especially under the low-K^+^ conditions.

### OsHAK8 Mediates K^+^ Transport in Yeast

**FIGURE 3:**
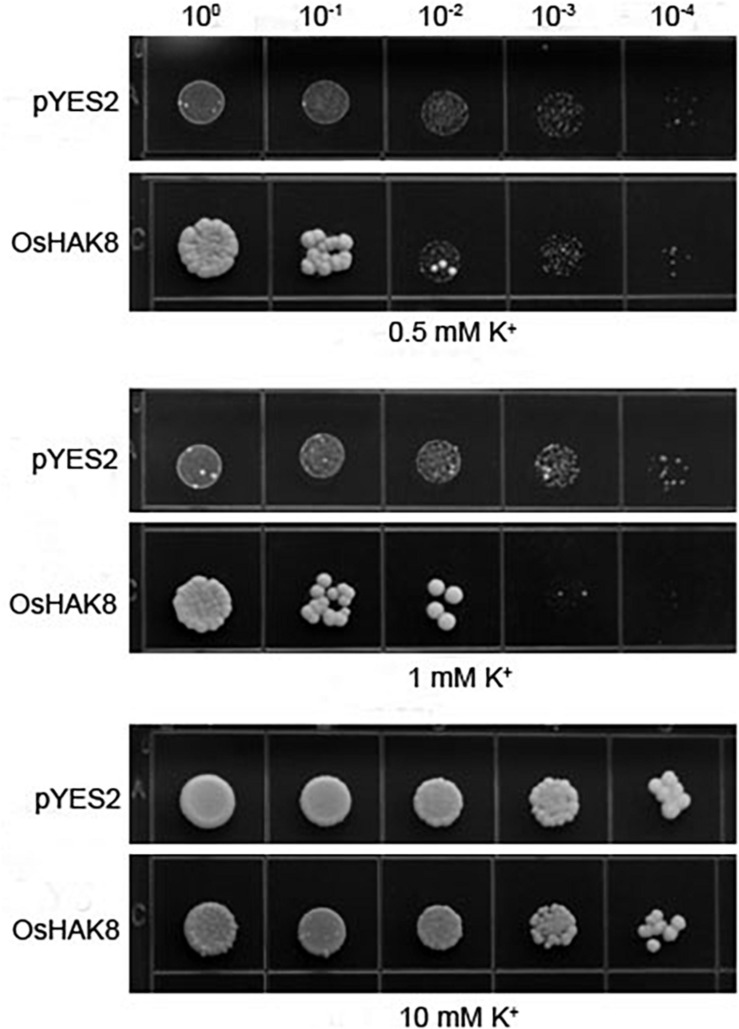
Functional complementation of *OsHAK8* in the K^+^ uptake-deficient mutant strain CY162 of *Saccharomyces cerevisiae*. Growth of yeast strains on arginine phosphate (AP) medium containing different K^+^ concentrations. The strains are CY162 cells transformed with the pYES2 vector only (pYES2), *OsHAK8* cDNA in the pYES2 vector (OsHAK8). CY162 cells which harbored pYES2 or expressed OsHAK8, were grown on the AP medium containing 0.5, 1, or 10 mM of K^+^. No significant differences were found between pYES2 and OsHAK8 when grown on the AP medium containing 10 mM of K^+^ (*P*>0.05 by Student’s *t*-test). Significant differences were found between pYES2 and OsHAK8 when grown on the AP medium containing 0.5 mM K^+^ (*P*<0.01 by Student’s *t*-test). The experiment was repeated three times with similar results.

To determine whether *OsHAK8* encodes a functional K^+^ transporter, we performed complementation tests using the yeast strain CY162 (*MAT*α, Δ*trk1*, *trk2*:pCK64, *his3*, *leu2*, *ura3*, *trp1*, and *ade2*), which lacks the high-affinity K^+^-transporters Trk1/2 and, thus, is defective in K^+^ uptake (Anderson et al., 1992). When grown on an arginine phosphate (AP) medium containing 10 mM of K^+^, no significant differences were scored among transformants harboring either the empty vector or the *OsHAK8* construct (Figure 3). When the concentration of K^+^ in the AP medium was decreased to 0.5 mM, the yeast mutant transformed with OsHAK8 grew better as compared to the mutant transformed with the empty pYES2 vector (Figure 3). The results indicated that OsHAK8 is capable of mediating K^+^ uptake.

### *OsHAK8* Encodes a Plasma Membrane Protein

**FIGURE 4:**
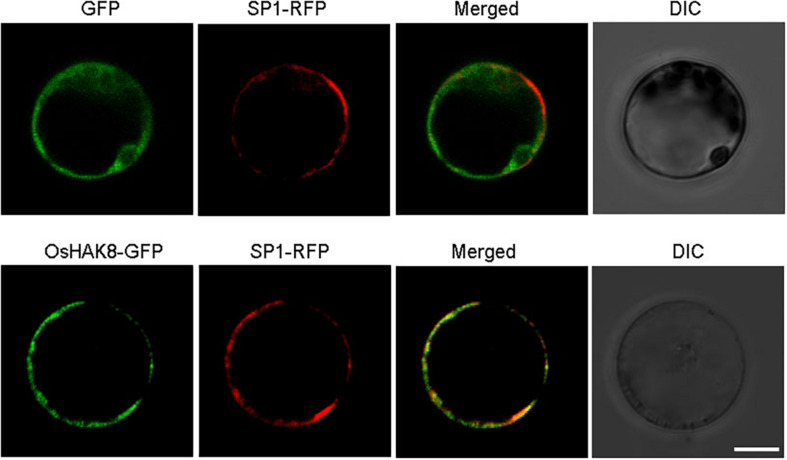
Plasma membrane localization of OsHAK8. The GFP coding sequence was fused to the C-terminus of the *OsHAK8* coding region in the pCambia2300 vector and then transformed into rice mesophyll protoplasts. GFP alone in the same vector was used as a control. Signals from GFP, OsHAK8-GFP, and SP1-RFP (a plasma membrane localization marker) were imaged using a Zeiss LSM880 confocal laser scanning microscope. Columns 1–4 show GFP signals, SP1-RFP signals, merged images of GFP and RFP signals, and bright-field differential interference contrast (DIC), respectively. For each localization experiment, ≥ 30 individual cells were analyzed using a Zeiss LSM880 confocal laser scanning microscope (Carl Zeiss). Bar = 10 μm.

The expression of *OsHAK8* facilitated K^+^ uptake in yeast, which suggested that OsHAK8 was likely to be targeted to the plasma membrane. To test the subcellular localization of OsHAK8 *in planta*, a green fluorescence protein (GFP) reporter construct was generated to express an OsHAK8-GFP fusion protein driven by the cauliflower mosaic virus 35S promoter. The same vector expressing GFP only was used as a control. The GFP-only control and the OsHAK8-GFP fusion construct were each introduced into the protoplasts from rice stem cells, and GFP signals were examined using confocal microscopy. While the GFP-only signal was observed mainly in the cytoplasm and nucleus, the OsHAK8-GFP fluorescence was predominantly localized at the plasma membrane. To confirm the plasma membrane localization of OsHAK8, we co-transformed the OsHAK8-GFP construct with a construct containing a known plasma membrane protein fused to red fluorescent protein (SP1-RFP) (Li et al., 2009) into the protoplasts from rice stem cells. We found that GFP signals produced by the OsHAK8-GFP fusion overlapped well with RFP signals generated by SP1-RFP (Figure 4). Based on these results, we concluded that OsHAK8 is localized to the plasma membrane in rice cells.

### *OsHAK8* Is Expressed Mainly in Rice Root

**FIGURE 5:**
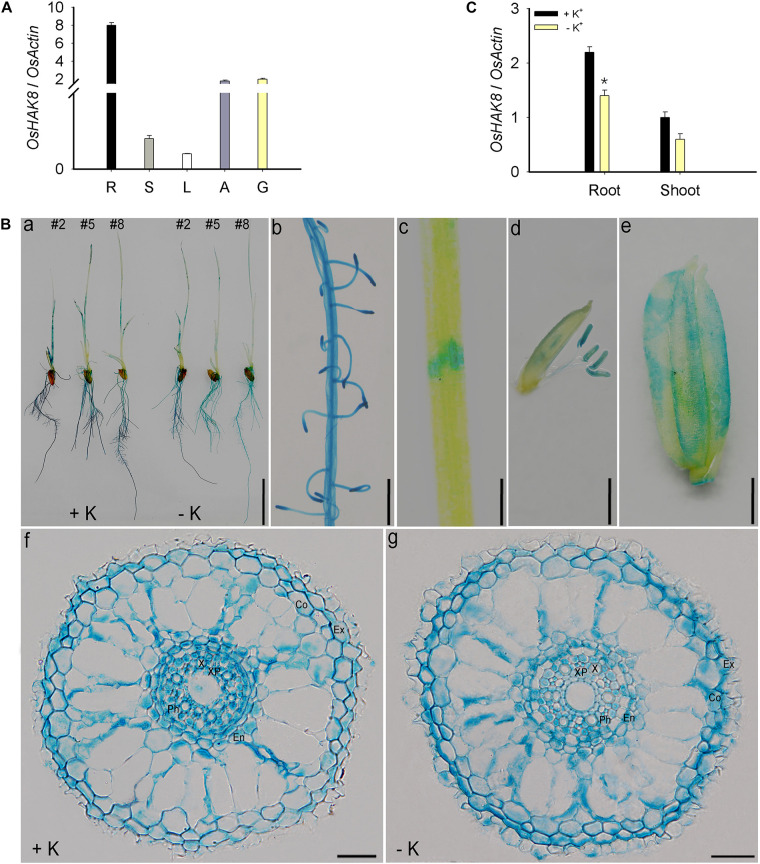
Expression pattern of *OsHAK8*. qRT-PCR analyses showing *OsHAK8* mRNA accumulation in roots, stems, leaves, anthers, and glumes. Rice seedlings (*Nipponbare*) were grown in soil for 10 weeks. R, root; S, stem; L, leaf; A, anther; G, glume. **(A)** Histochemical analysis of *OsHAK8* promoter-driven GUS reporter expression in transgenic rice plants. The transgenic plants were grown as described in **(B)**, and GUS activities (indicated in blue) were examined using histochemical staining. (a) GUS staining of 7-days-old plants (Line 2) grown in hydroponic solutions with 10-mM K^+^ (+ K) or 0-mM K^+^ (-K). (b) Enlarged image of the root hair zones in (a). (c) Enlarged image of the node in (a). (d) GUS staining of anthers. The transgenic plants were grown as described in **(A)**. (e) GUS staining of glume. The transgenic plants were grown as described in **(A)**. (f,g) Cross-section images of the elongation zone in (a). Ex, exodermis; Co, cortex; En, endodermis; Ph, phloem; X, xylem; XP, xylem parenchyma. Bar in (a) = 1 cm, bar in (b) = 5 mm, bar in (c) = 1 mm, bar in (d) = 1 mm, bar in (e) = 1 mm, and bars in (f) to (g) = 100 μm. **(C)** Effect of K^+^ deficiency on the transcriptional expression of *OsHAK8* in rice. 3-day-old rice seedlings (*Nipponbare*) were grown in a hydroponic solution containing 10 mM K^+^ for 3 days and then transferred to 10-mM K^+^ (+ K) and 0-mM K^+^ (−K) solutions for 24 h, respectively. Total RNAs were isolated from the roots and shoot of the seedlings, and *OsHAK8* transcript levels were determined by qRT-PCR. *Actin* was used as an internal standard. Significant difference was found between 0- and 10-mM K^+^ samples indicated in root tissue (*P*<0.01 by Student’s *t*-test). Data are means of three replicates of one experiment. The experiment was repeated three times with similar results. Error bars represent ± SD. Asterisks represent significant differences.

Analyses of the *OsHAK8* expression pattern are critical to explaining the physiological functions of the gene in plants. We first determined the expression pattern of the *OsHAK8* gene in different tissues of rice plants. The qRT-PCR analysis of the RNA samples isolated from various organs of the *Nipponbare* plants showed that *OsHAK8* was expressed mainly in the roots. Transcripts of *OsHAK8* were also present in lower amounts in stems, leaves, anthers, and glumes (Figure 5A). To investigate the expression pattern of *OsHAK8* in more detail, we performed GUS activity staining of the transgenic rice plants harboring the *OsHAK8* promoter-GUS fusion construct. Consistent with the qRT-PCR results, strong GUS signals were detected in the roots (Figures 5Ba,b). In addition, GUS activity was also detected in the stem (Figure 5Bc), mature anthers (Figure 5Bd), and glume (Figure 5Be). We chose three independent transgenic lines (lines 2, 5, and 8) to analyze the results in more detail through the examination of root cross-sections. The results showed that *OsHAK8*-GUS was expressed mainly in the vascular tissues, cortex, and exodermis of the roots (Figure 5Bf).

Expression analysis showed that the expression of *OsHAK8* in the roots was downregulated when seedlings were transferred from the K^+^-sufficient to the K^+^-deficient condition (Figure 5C). In addition, the *OsHAK8*-GUS signal was decreased by K^+^ deficiency in stelar cells when transferred to K^+^-deficient conditions (Figures 5Ba,f,g and Supplementary Figure 4A). We quantified the GUS signals in the root stele using the Image J software in three plants from lines (lines 2, 5, and 8) treated under K^+^-sufficient or K^+^-deficient conditions. There was a 2-fold decrease in the GUS signal in the stele under K^+^-deficient conditions (Supplementary Figure 4B). This expressed pattern suggests the potential role of *OsHAK8* in K^+^ transport in response to the low-K^+^ stress in the roots.

### Root Uptake and Root-to-Shoot K^+^ Transport Are Reduced in *Oshak8* Mutants Under K^+^-Deficient Condition

The expression analysis indicated that *OsHAK8* showed strong expression in root tissues (Figure 5A). Direct K^+^ measurements indicated that the roots and shoots of the two *Oshak8* mutants both accumulated less K^+^ than that of the wild-type plants under low-K^+^ conditions (Figures 2B,C). These results suggest that *OsHAK8* may be involved in K^+^ acquisition and/or root-to-shoot translocation in root tissues.

**FIGURE 6:**
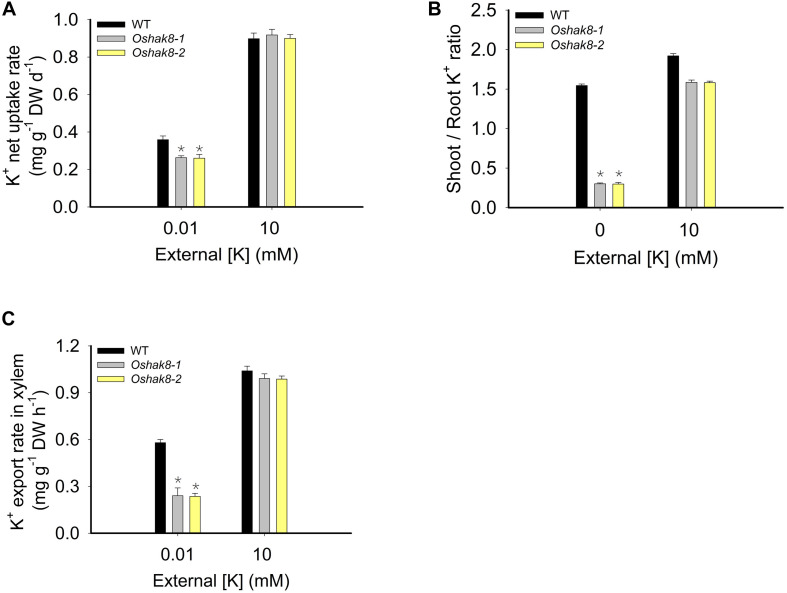
Effects of *Oshak8* knockout on K translocation under different K^+^-supply conditions. **(A)** K^+^ net uptake rate of WT and *Oshak8* mutant (*Oshak8*-1, *Oshak8*-2) plants. The seeds of the WT and *Oshak8* mutants were germinated in water for 5 days, transferred to the hydroponic solutions containing 10 mM of K^+^ for 14 days, and then transferred to the hydroponic solutions containing 0 mM of K^+^ for 5 days. For the K^+^ uptake assay, K^+^-starved plants were incubated in the medium containing 0.01 or 10 mM of K^+^ for 3 days and the roots and shoots were collected for K^+^ content measurements using inductively coupled plasma analysis. **(B)** The shoot/root ratios of K^+^ in various plant materials. Growth conditions were as described in Figure 2. The roots were collected for K^+^ content measurements using inductively coupled plasma analysis. **(C)** K^+^ export rate in the xylem. Seven-day-old rice seedlings were grown in the International Rice Research Institute (IRRI) solution for 2 weeks and then transferred to 10- or 0.01-mM K^+^ solutions for 2 weeks before the sampling. The detailed protocols for the shoot excision, collection of the xylem sap by cotton ball, determination of K^+^ concentration by ICP, and calculation of the xylem K^+^ export rate were described previously by Yang et al. (2014). K^+^ contents were determined by ICP-AES. Significant differences were found between WT and *Oshak8* mutants (*Oshak8-1*, *Oshak8-2*) (*P < 0.01 by Student’s *t*-test). Data are means of three replicates of one experiment. The experiment was repeated three times with similar results. Error bars represent ± SD. Asterisks represent significant differences. DW, dry weight.

To test whether *OsHAK8* participated in K^+^ uptake, we performed the K^+^ uptake assay. The seeds of the wild-type plants and *Oshak8* mutants were germinated in water for 5 days, transferred to the hydroponic solutions containing 10 mM of K^+^ for 14 days, and then transferred to the hydroponic solutions containing 0 mM of K^+^ for 5 days. For the K^+^ uptake and translocation assay, K^+^-starved plants were incubated in the medium containing 0.01 or 10 mM of K^+^ for 3 days, and the roots and shoots were collected separately for K^+^ content measurements using ICP analysis. As shown in Figure 6A, the K^+^ uptake rate was reduced in the *Oshak8* mutants compared with the wild-type plants. The results showed that the knockout of *OsHAK8* impaired K^+^ uptake in rice roots.

We calculated the shoot/root ratio of K^+^ content to determine the K^+^ distribution between shoots and roots. The shoot/root K^+^ ratio in the *Oshak8* mutants was lower than that of the wild-type plants under K^+^-deficient conditions (Figure 6B), indicating that K^+^ translocation from roots to shoots was affected in *Oshak8* mutants. As a critical parameter for root-to-shoot transport, K^+^ concentration in the xylem sap was also measured. Under K^+^-sufficient conditions (10 mM of K^+^), there was no difference in K^+^ levels in the xylem sap between mutants and wild-type plants (Figure 6C). Under low-K^+^ conditions (0.01 mM of K^+^), the K^+^ levels in the xylem sap were lower in *Oshak8* compared to the wild-type (Figure 6C), consistent with the reduced root-to-shoot K^+^ transport in the *Oshak8* mutants. Taken together, these results demonstrated that *OsHAK8* is involved in K^+^ uptake and root-to-shoot K^+^ transport in roots, especially under low-K^+^ conditions.

## Discussion

Although OsAKT1 and OsHAK5, like AtAKT1 and AtHAK5 in *Arabidopsis*, mediate K^+^ uptake in rice roots (Li et al., 2014; Yang et al., 2014), more transporters related to rice K^+^ absorption and translocation need to be explored. Here, we showed that the *OsHAK* family gene *OsHAK8* plays a crucial role in K^+^ uptake and root-to-shoot K^+^ transport in rice roots.

The GUS reporter analysis indicated that *OsHAK8* was broadly expressed in different cell types in the roots (Figure 5Cf), especially in the exodermis, cortex, and stele (Figures 5Cb,f), which support the possible function of the gene in K^+^ uptake and root-to-shoot K^+^ translocation in rice roots. Several lines of genetic data connected growth defects of the *Oshak8* mutant plants with the K^+^ transport function of the OsHAK8. The K^+^ contents in both the roots and shoots of the *Oshak8* mutants were significantly reduced (Figures 2B,C), suggesting that the growth retardation of *Oshak8* may be due to the reduction of K^+^ uptake and root-to-shoot K^+^ transport. Furthermore, the results from K^+^ depletion assays in which both root uptake and root-to-shoot K^+^ transport were measured again indicated that the *Oshak8* mutants performed worse as compared to the wild-type plants. As K^+^ is a critical nutrient that supports plant growth, reduced K^+^ uptake and root-to-shoot K^+^ transport in the *Oshak8* mutants may result in the inhibition of plant growth in both young seedlings and adult plants in the field (Figure 1A and Supplementary Figures 2A,B). Another process where OsHAK8 may function is in gametogenesis associated with pollen viability and seed setting. We observed reduced pollen viability and 1,000-grain weight associated with the mutant plants (Supplementary Figures 2E, 3A,B). Together, these results show that OsHAK8 is expressed and functions in multiple tissues and developmental stages in rice plants. Because OsHAK8 is a plasma membrane transporter, its function in K^+^ uptake may be due to its expression and activity in the root epidermal cells. In addition, its expression in the stelar cells may contribute to K^+^ transport into the vasculature and, eventually, secretion into the xylem vessels. Detailed analyses of K^+^ contents in various cell types of vascular tissues may help further dissect the role of OsHAK8 in a specific step of root-to-shoot long-distance transport.

Plants can sense K^+^ levels in the soil and accordingly regulate root-to-shoot translocation to balance K^+^ distribution between roots and shoots (Du et al., 2019). Under K^+^-sufficient conditions, the root-to-shoot K^+^ transport is enhanced to support plant growth. In contrast, when facing K^+^ deficiency in the soil, the long-distance transport activity is often reduced to maintain root growth as a priority. In other words, root-to-shoot K^+^ transport needs to be fine-tuned according to external K^+^ levels (Du et al., 2019). Transcriptional regulation provides an essential strategy in plant regulation of root-to-shoot translocation. Previous studies have revealed that the NRT1/PTR family member *NPF7.3/NRT1.5* is regulated at the transcriptional level in response to external low-K^+^ levels. During low-K^+^ stress, *NPF7.3/NRT1.5* transcripts are downregulated to inhibit root-to-shoot K^+^ transport in *Arabidopsis* (Du et al., 2019), which suggests that root-to-shoot K^+^ translocation can be achieved *via* the transcriptional regulation of related transporters such as NPF7.3/NRT1.5. In the present study, we found that *OsHAK8* functions in root-to-shoot transport, especially under low-K^+^ conditions. Interestingly, *OsHAK8* transcript level was reduced in response to K^+^ deficiency in root stelar cells (Figures 5Ba,g,C and Supplementary Figures 4A,B), consistent with the idea that downregulation of root-to-shoot K^+^ translocation may be achieved through reduced *OsHAK8* expression. However, downregulation of *OsHAK8* expression under low-K^+^ conditions does not mean plants do not need them. In loss-of-function mutants of *OsHAK8*, root-to-shoot K^+^ transport was largely abolished (Figure 6C), causing stunted growth (Figure 1A and Supplementary Figure 2Aa). Therefore, the transcript levels of *OsHAK8* need to be fine-tuned in response to external K^+^ levels. These transcriptional levels of *OsHAK8* may be regulated by some yet unknown components in the low-K^+^ response pathway. This hypothesis should be investigated in future experiments.

Compared with *Arabidopsis HAK* members, the rice genome features a large gene expansion in the *HAK* family. The dramatic gene expansion events in the rice *HAK* family imply that K^+^ uptake and transport mechanisms in rice are more complex. Indeed, according to the expression patterns in rice roots, different OsHAK transporters are expressed and responsible for K^+^ transport in different root cell types in response to external low K^+^ levels. For example, *OsHAK1* is abundantly expressed in entire roots, particularly at the root tips. *OsHAK5* is much less abundant in roots as compared to shoots. The expression of *OsHAK16* is detected at the root epidermal and stelar cells. *OsHAK8* was abundantly expressed in almost all cell types of roots. K^+^ deficiency steadily upregulated the expression of *OsHAK1* and *OsHAK16* in the roots, while enhancing *OsHAK5* expression only transiently (Yang et al., 2014; Chen et al., 2015; Feng et al., 2019). In contrast, we found that K^+^ deficiency downregulated the expression of *OsHAK8* in the steles. Such unique expression patterns of different *OsHAK* genes may have determined their functions in plants. *OsHAK5* is involved in the root K^+^ acquisition and root-shoot translocation only at low (0.1–0.3 mM), but not at high (1 mM) external K^+^ mediums (Yang et al., 2014). *OsHAK1* and *OsHAK16* contribute to K^+^ acquisition and root-shoot translocation in the 0.05–1 mM range. Here, we showed that *OsHAK8* mediated K^+^ acquisition and root-shoot translocation at a wide range of external K^+^ levels (0.01–10 mM). For further dissection of the distinct roles of OsHAK8, OsHAK1, OsHAK5, and OsHAK16 in rice, it will be important to reveal the regulatory mechanisms of OsHAK1, OsHAK5, OsHAK16, and OsHAK8 at both the transcriptional and post-transcriptional levels in the future.

## Data Availability Statement

The datasets presented in this study can be found in online repositories. The names of the repository/repositories and accession number(s) can be found in the article/Supplementary Material.

## Author Contributions

XW, DM, LC, and SL conceived, designed the experiments, and analyzed the data. XW, JL, FL, DC, and YP performed the experiments. XW, DM, and SL wrote the manuscript. All authors contributed to the article and approved the submitted version.

## Conflict of Interest

The authors declare that the research was conducted in the absence of any commercial or financial relationships that could be construed as a potential conflict of interest.

## Publisher’s Note

All claims expressed in this article are solely those of the authors and do not necessarily represent those of their affiliated organizations, or those of the publisher, the editors and the reviewers. Any product that may be evaluated in this article, or claim that may be made by its manufacturer, is not guaranteed or endorsed by the publisher.

## Footnotes

1
http://dsdecode.scgene.com/

2
http://cbi.hzau.edu.cn/cgi-bin/CRISPR

## Supplementary Material

Supplementary Figure 1 Verification of the *Oshak8* mutants. Verification of the *Oshak8* mutants by PCR-based sequencing. Two representative transgenic lines (abbreviated as *Oshak8-1* and *Oshak8-2*, respectively) for *Oshak8* mutants are generated from the *Nipponbare* genetic background.

Supplementary Figure 2 Phenotype analysis of *Oshak8* mutants grown in soil. **(A)** Phenotype analysis of plants grown in soil. (a) Phenotype of plants grown in soil. WT and *Oshak8* mutants (*Oshak8-1*, *Oshak8-2*) grown in soil (0.18-mg/g K^+^) for 95 days. Bar = 20 cm. (b) Comparison of brown spots, which is a typical K^+^-deficient symptom of rice on old leaves of plants. Growth conditions were as described in **(A)**. *Oshak8-1* and *Oshak8-2* displayed brown spots on old leaves. WT displayed no brown spots on old leaves. Bar = 1 cm. (c) The main panicle lengths of plants. WT and *Oshak8* mutants (*Oshak8-1*, *Oshak8-2*) grown in soil (0.18-mg/g K^+^) for 105 days. Main panicle lengths were measured. Bars = 8 cm. Data are presented as means ± SD. Bar = 10 cm. **(B–E)** Statistical analysis of plant heights **(B)**, main panicle length **(C)**, seed setting rate **(D)**, and 1,000-grain weight **(E)** of plants. Growth conditions were as described in **(A)**. Significant differences were found between WT and *Oshak8* mutants (*Oshak8-1*, *Oshak8-2*) (^^*^*P* < 0.01 by Student’s *t*-test). *n* ≥ 20 for each data point. Asterisks represent significant differences.

Supplementary Figure 3 Pollen viability analysis of *Oshak8* mutants. **(A)** I_2_-KI staining of pollen grains from the wild type and *Oshak8* mutants (*Oshak8-1*, *Oshak8-2*). Bar = 100 μm. **(B)** Pollen viability of the wild-type and *Oshak8* mutants (*Oshak8-1*, *Oshak8-2*). The experiment was repeated three times with similar results. Error bars represent ± SD. Significant difference was found between wild-type and *Oshak8* mutants (*Oshak8-1*, *Oshak8-2*) (*P* < 0.01 by Student’s *t*-test). The experiment was repeated three times with similar results. Error bars represent ± SD.

Supplementary Figure 4 Quantitative analysis of *OsHAK8* promoter activity represented by GUS signal. Histochemical analysis of *OsHAK8* promoter-driven GUS reporter expression in transgenic rice plants. Three-day-old rice seedlings (*Nipponbare*) were grown in hydroponic solution containing 10 mM K^+^ for 3 days and then transferred to 10 mM K^+^ (+ K) and 0 mM K^+^ (-K) solutions for 24 h, respectively. GUS activity was examined by staining with GUS substrate (X-Gluc) in three independent lines (#2, #5, #8) of transgenic plants harboring *OsHAK8*-GUS construct. Plants were stained in each line and a representative plant was shown in each group. **(A)** Cross-section images of the root hair zone of the transgenic plants harboring *OsHAK8*-GUS construct (#2, #5, #8) in **(A)**. Bars = 100 μm. **(B)** Quantification of GUS activity in the cortex region of transgenic rice plants containing the *OsHAK8* promoter-GUS construct in **(A)**. Image J software was used to quantify the GUS signals in the root cortex areas of the transgenic lines (#2, #5, #8) treated with K^+^-sufficient (10-mM K^+^) or low-K^+^ conditions (0-mM K^+^). Three plants from each line were used for the analysis in one experiment. The experiment was repeated three times with similar results. Error bars represent ± SD. Significant difference was found between 0.01- and 10-mM K^+^ samples are indicated in root tissues (*P* < 0.01 by Student’s *t*-test). The experiment was repeated three times with similar results. Error bars represent ± SD.

Supplementary Table 1 List of PCR primers.
