# Supplementary material for: Rice Potassium Transporter OsHAK8 Mediates K+ Uptake and Translocation in Response to Low K+ Stress
Source: Front Plant Sci. 2021 Aug 3;12:730002. doi: 10.3389/fpls.2021.730002 (PMC8369890; doi:10.3389/fpls.2021.730002)
Supplement: Supplementary Figure 1 — Verification of the Oshak8 mutants. Verification of the Oshak8 mutants by PCR-based sequencing. Two representative transgenic lines (abbreviated as Oshak8-1 and Oshak8-2, respectively) for Oshak8 mutants are generated from the Nipponbare genetic background. [file Data_Sheet_1.docx]

**Supplemental datas**


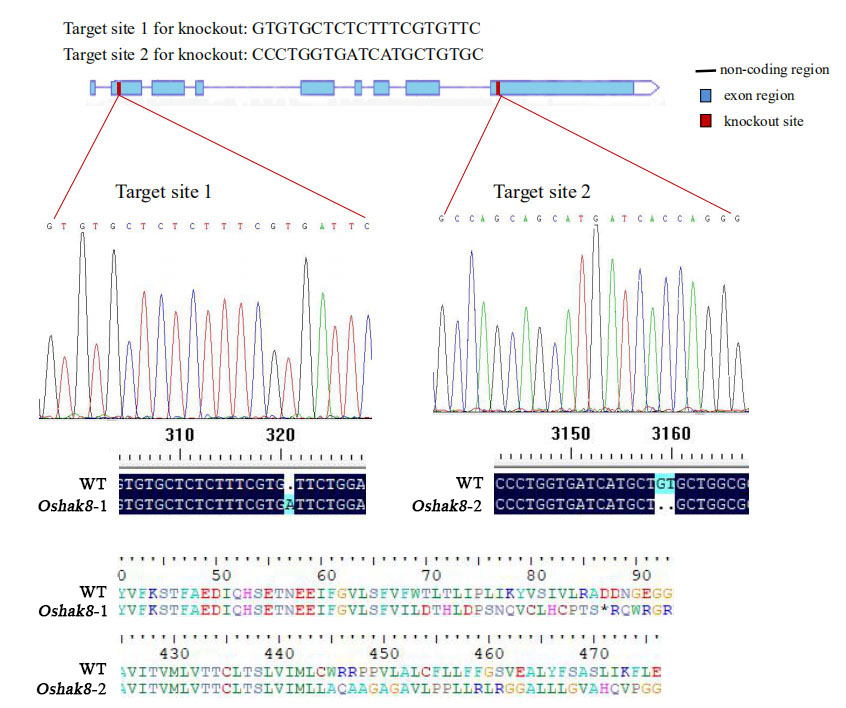


**Figure S1. Verification of the *Oshak8* mutants.**

Verification of the *Oshak8*mutants by PCR-based sequencing. Two representative transgenic lines (abbreviated as *Oshak8-1* and *Oshak8-2*, respectively) for *Oshak8* mutants are generated from *Nipponbare* genetic background.


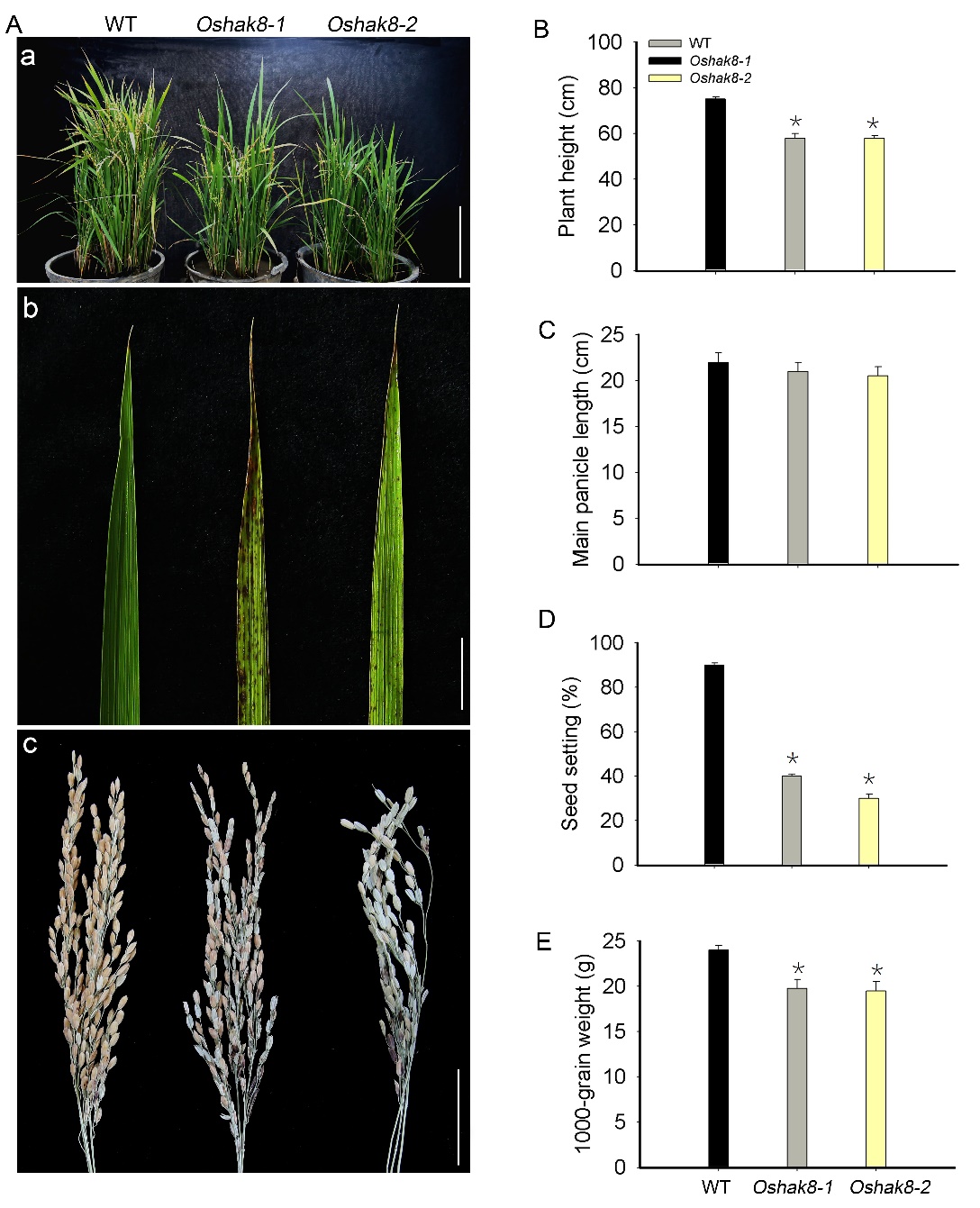


**Figure S2. Phenotype analysis of *Oshak8* mutants grown in soil.**

(A) Phenotype analysis of plants grown in soil. (a) Phenotype of plants grown in soil. The WT and *Oshak8* mutants (*Oshak8-1*,*Oshak8-2*) grown in soil (0.18 mg/g K^+^ ) for 95 days. Bar = 20 cm.(b) Comparison of brown spots which was a typical K^+^-deﬁcient symptom of rice on old leaves of plants. Growth conditions were as described in Figure S2A.*Oshak8-1* and *Oshak8-2* displayed brown spots on old leaves. WT displayed no brown spots on old leaves. Bar = 1 cm.(c) The main panicle lengths of plants. WT and *Oshak8* mutants (*Oshak8-1*,*Oshak8-2*) grown in soil (0.18 mg/g K^+^ ) for 105 days. Main panicle lengths were measured. Bars = 8 cm. Data are presented as means ± SD. Bar = 10 cm.

(B-E) [Statistical analysis](https://www.baidu.com/link?url=ixYeEdOgycmg0jXFUKLfzwdqINUEEG4ttK0f_XAdRq3KD4wktQKAw4NIIT9HCwd-y4so_L-kk9atSGUrvRSSsdLwu3RHc4vqHIk3KX59VySkw3zc8r1MNs_q070n5jNB&wd=&eqid=ef15c8720000014b000000065f2a5f83) of plant heights (B), Main panicle length (C), seed setting rate (D) and 1000 -grain weight (E) of plants. Growth conditions were as described in Figure S2A. Signiﬁcant differences were found between WT and *Oshak8* mutants (*Oshak8-1*, *Oshak8-2*) (^＊^P < 0.01 by Student’s t test). n ≥ 20 for each data point. Asterisks represent significant differences.


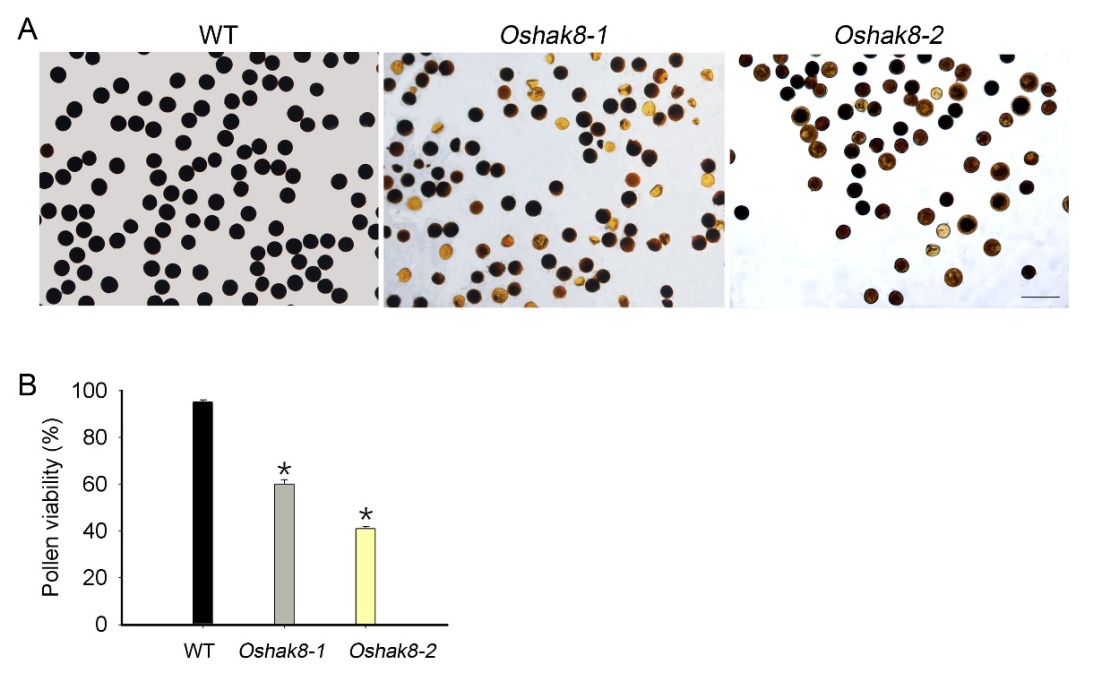


**Figure S3. Pollen viability analysis of *Oshak8* mutants.**

(A) I_2_-KI staining of pollen grains from the wild type and *Oshak8* mutants (*Oshak8-1*, *Oshak8-2*). Bar=100μm.

(B) Pollen viability of the wild type and *Oshak8* mutants (*Oshak8-1*, *Oshak8-2*).

The experiment was repeated three times with similar results. Error bars represent ±SD. Signiﬁcant difference was found between wild type and *Oshak8* mutants (*Oshak8-1*, *Oshak8-2*) (P< 0.01 by Student’s t test). The experiment was repeated three times with similar results. Error bars represent ±SD.


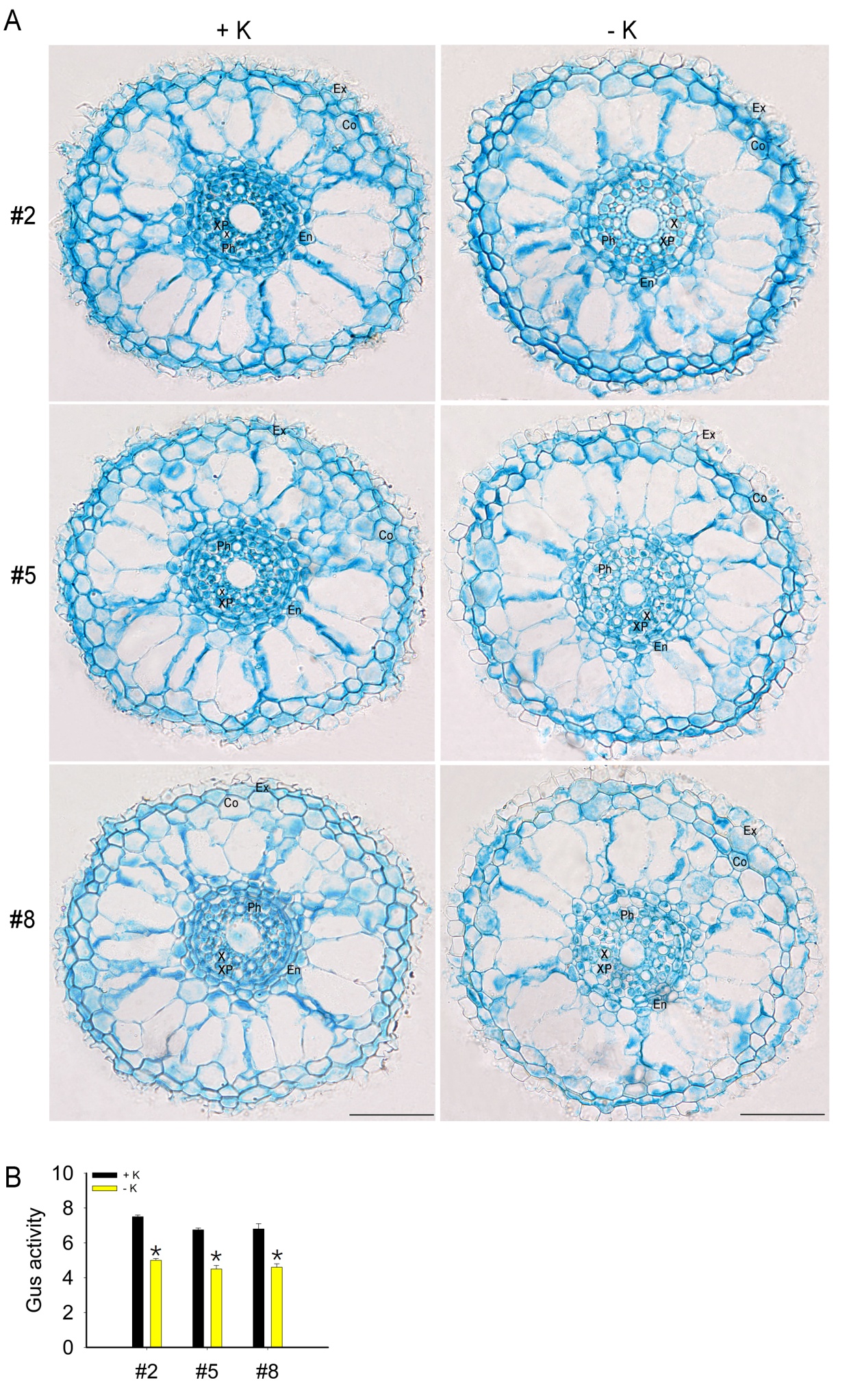


**Figure S4. Quantitative Analysis of *OsHAK8* Promoter Activity Represented by GUS Signal.**

Histochemical analysis of *OsHAK8* promoter-driven GUS reporter expression in transgenic rice plants. 3-d-old rice seedlings (*Nipponbare*) were grown in hydroponic solution containing 10 mM K^+^ for 3 d and then transferred to 10 mM K^+^ (+ K) and 0 mM K^+^ (- K) solutions for 24 h, respectively. GUS activity was examined by staining with GUS substrate (X-Gluc) in three independent lines (#2, #5, #8) of transgenic plants harboring *OsHAK8*-GUS construct. Plants were stained in each line and a representative plant was shown in each group.

(A) Cross section images of the root hair zone of the transgenic plants harboring *OsHAK8*-GUS construct (#2, #5, #8 ) in (Figure S4A). Bars = 100 µm.

(B) Quantiﬁcation of GUS activity in the cortex region of transgenic rice plants containing the *OsHAK8* promoter-GUS construct in (Figure S4A). Image J software was used to quantify the GUS signals in the root cortex areas of the transgenic lines (#2, #5, #8) treated with K^+^-sufﬁcient (10 mM K^+^) or low-K^+^ conditions (0 mM K^+^). Three plants from each line were used for the analysis in one experiment. The experiment was repeated three times with similar results. Error bars represent ±SD. Signiﬁcant difference was found between 0.01 and 10 mM K^+^ samples are indicated in root tissues (P< 0.01 by Student’s t test). The experiment was repeated three times with similar results. Error bars represent ±SD.

**Table S1. List of PCR Primers**

| Name | Primer Sequences | Purposes |
| --- | --- | --- |
| Cas9-1-OsHAK8F | GGCAGTGTGCTCTCTTTCGTGTTC | CRISPR/Cas9 construction |
| Cas9-1-OsHAK8R | AAACGAACACGAAAGAGAGCACAC |  |
| Cas9-2-OsHAK8F | GGCACCCTGGTGATCATGCTGTGC | CRISPR/Cas9 construction |
| Cas9-2-OsHAK8R | AAACGCACAGCATGATCACCAGGG |  |
| OsHAK8-CDS-F | ATGGATCTTGAGTTTGGGAG | Cloning of *HAK8* CDNA |
| OsHAK8-CDS-R | TCATAGCACGTAGACCATGCC |  |
| p1301-HAK8X1F | GAGTCCACTCGCAAGGTCG | Cloning of *HAK8* promoter |
| p1301-HAK8X1R | TGCTCGGCCAAGAACCTAT |  |
| HAK8-GFP- F | ggtacccggggatcctctagaATGGATCTTGAGTTTGGGAG | Subcellular localization construction |
| HAK8-GFP- R | agctcctcctcctcctctagaTAGCACGTAGACCATGCC |  |
| PYES2-HAK8F | actatagggaatattaagcttATGGATCTTGAGTTTGGGAG | CY162 complementation construct of *HAK8* |
| PYES2-HAK8R | tacatgatgcggccctctagaTCATAGCACGTAGACCATGCC |  |
| qRT HAK8-F | GGGACTCATGGAAGACAACC | Q-PCR analysis of *HAK8* expression pattern |
| qRT HAK8-R | GAACACGAAAGAGAGCACAC |  |
| Actin-F | CAATGTGCCAGCTATGTATGTCGCC | Q-PCR analysis of Actin |
| Actin-R | TTCCCGTTCAGCAGTGGTAGTGAAG |  |
